# Monitoring of the Village Malaria Workers to conduct activities of Malaria Elimination Demonstration Project in Mandla, Madhya Pradesh

**DOI:** 10.1186/s12936-021-04040-2

**Published:** 2022-01-08

**Authors:** Harsh Rajvanshi, Praveen K. Bharti, Ravendra K. Sharma, Sekh Nisar, Kalyan B. Saha, Himanshu Jayswar, Ashok K. Mishra, Aparup Das, Harpreet Kaur, Altaf A. Lal

**Affiliations:** 1 Malaria Elimination Demonstration Project, Mandla, Madhya Pradesh India; 2 Indian Council of Medical Research-National Institute of Research in Tribal Health (ICMR-NIRTH), Jabalpur, Madhya Pradesh India; 3 Indian Council of Medical Research-National Institute of Medical Statistics (ICMR-NIMS), New Delhi, India; 4 Directorate of Health Services, Government of Madhya Pradesh, Bhopal, India; 5 Indian Council of Medical Research, Department of Health Research, Ministry of Health and Family Welfare, New Delhi, India; 6 Foundation for Disease Elimination and Control of India, Mumbai, Maharashtra India; 7 Present Address: Asia Pacific Leaders Malaria Alliance (APLMA), Helios, Singapore; 8 Present Address: Indian Council of Medical Research-National Institute of Malaria Research (ICMR-NIMR), New Delhi, India; 9 Present Address: State Vector Borne Disease Control Programme, Raigarh, Chattisgarh India; 10 Ch. Charan Singh University, Meerut, Uttar Pradesh India

**Keywords:** Monitoring checklist, Malaria elimination, Operational accountability, Programme management

## Abstract

**Background:**

The capacity of the field staff to conduct activities related to disease surveillance, case management, and vector control has been one of the key components for successfully achieving malaria elimination. India has committed to eliminate malaria by 2030, and it has placed significance on monitoring and evaluation at the district level as one of the key strategies in its national framework. To support and guide the country’s malaria elimination objectives, the Malaria Elimination Demonstration Project was conducted in the tribal district of Mandla, Madhya Pradesh. Robust monitoring of human resources received special attention to help the national programme formulate a strategy to plug the gaps in its supply chain and monitoring and evaluation systems.

**Methods:**

A monitoring tool was developed to test the capabilities of field workers to conduct activities related to malaria elimination work. Between November 2018 to February 2021, twenty-five Malaria Field Coordinators (MFCs) of the project utilized this tool everyday during the supervisory visits for their respective Village Malaria Workers (VMWs). The data was analysed and the scores were tested for variations against different blocks, educational status, duration of monitoring, and post-training scores.

**Results:**

During the study period, the VMWs were monitored a total of 8974 times using the monitoring tool. Each VMW was supervised an average of 1.8 times each month. The critical monitoring indicators scored well in all seven quarters of the study as monitored by the MFCs. Monitoring by MFCs remained stable at 97.3% in all quarters. Contrary to expectations, the study observed longer diagnosis to treatment initiation time in urban areas of the district.

**Conclusion:**

This study demonstrated the significance of a robust monitoring tool as an instrument to determine the capacity of the field workers in conducting surveillance, case management, and vector control related work for the malaria elimination programme. Similar tools can be replicated not only for malaria elimination, but other public health interventions as well.

**Supplementary Information:**

The online version contains supplementary material available at 10.1186/s12936-021-04040-2.

## Background

Monitoring and evaluation is a key component to assess the progress and outcomes of specific interventions. Availability of high-quality and reliable disease burden data is imperative for making evidence-based decisions for programme officials at local levels and policymakers at state and national levels. There is interest and commitment to advance global malaria elimination efforts. To achieve that, a need for robust monitoring and evaluation has been highlighted by the World Health Organization [1, 2], African Leaders Malaria Alliance (ALMA) [3], Elimination Eight Initiative (E8) [4], and The Lancet Commission [5].

The malaria elimination efforts in Algeria [6], Timor-Leste [7], Sri Lanka [8], China [9], and Azerbaijan [10] placed priority on sound monitoring. From 2010 to 2019, the Asia–Pacific region has shown good progress towards malaria elimination by all the countries except Papua New Guinea, Afghanistan, and the Solomon Islands, where there has been an increase in malaria cases by 11%, 46%, and 80%, respectively [1]. The weak monitoring systems of these three countries were attributed to this surge [11–13]. The existing and new tools for malaria elimination will be only effective under a net of sound monitoring, learning, and feedback mechanisms.

India has also pledged its commitment to eliminate malaria by 2030 with a strong focus on monitoring and evaluation of surveillance, vector control, Information Education Communication and Behaviour Change Communication (IEC and BCC), and capacity building from district to national-level [14]. Complementing the National Vector Borne Disease Control Programme’s (NVBDCP) efforts to eliminate malaria from India, the Malaria Elimination Demonstration Project’s (MEDP) goal was to demonstrate that malaria elimination is possible in areas with varying Annual Parasite Incidences (APIs) in 1233 villages of Mandla district of Madhya Pradesh.

MEDP is a public–private partnership between the Government of Madhya Pradesh, the Indian Council of Medical Research (ICMR) through the National Institute of Tribal Health (ICMR-NIRTH), and the Foundation for Disease Elimination and Control of India, which was established by Sun Pharmaceutical Industries Ltd. as a not-for-profit entity. MEDP started its field activities in April 2017. The project developed a Monitoring and Evaluation (M&E) framework in consultation with the ICMR—National Institute of Epidemiology (ICNR-NIE) [15]. This framework described the key requirements in the monitoring of the elimination project, which gave birth to the monitoring tool. This paper aims to develop and demonstrate a replicable and sustainable monitoring strategy of front-line health staff. This strategy is expected to plug the identified gaps of sustenance of an effective supply chain systems and lack of robust M&E system listed in the National Strategic Plan for Malaria Elimination of the National Vector Borne Disease Control Programme, Government of India [16].

## Methods

### Study area

**Fig. 1:**
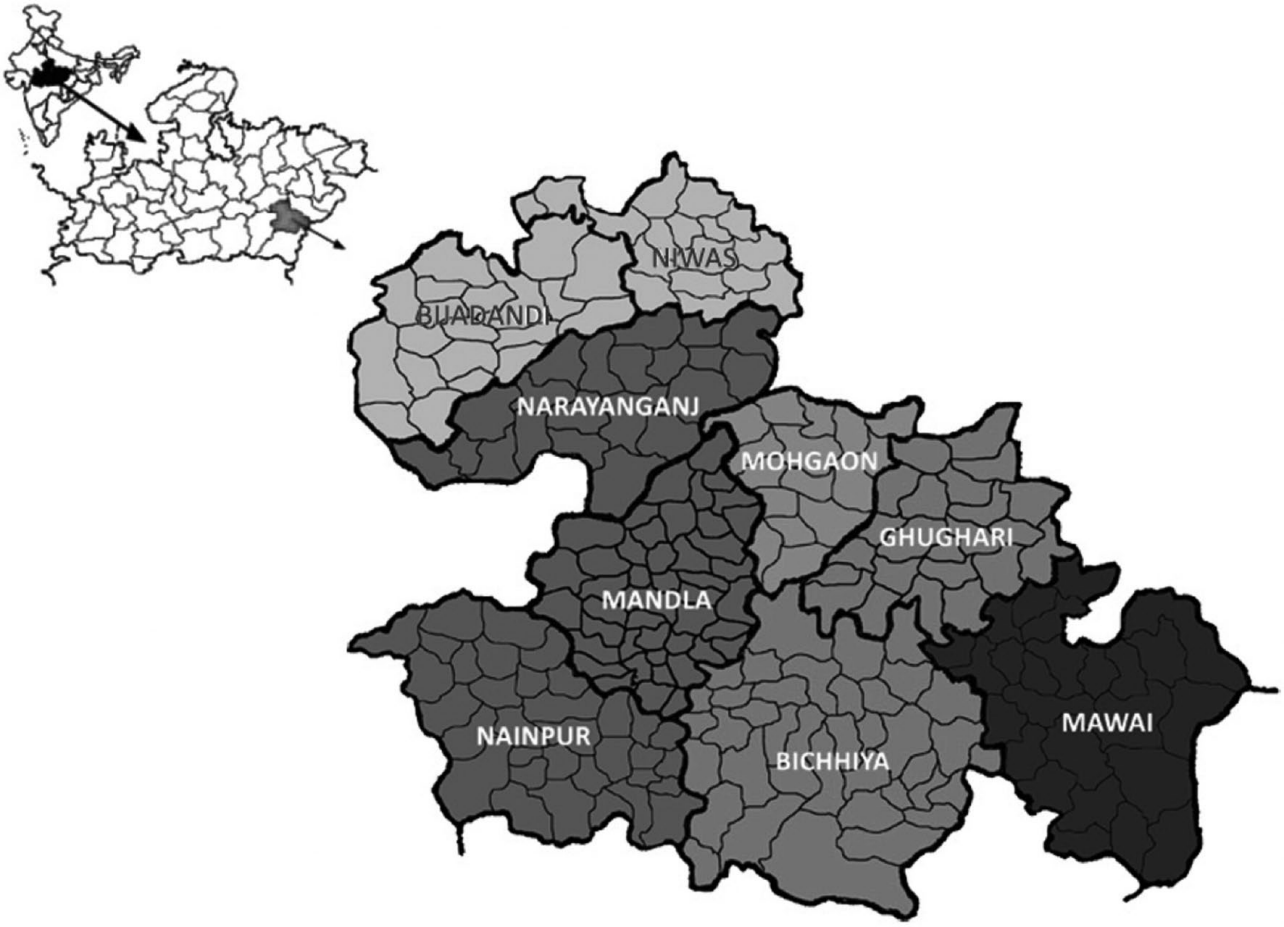
Blown out map (India–Madhya Pradesh–Mandla) of the study district with all the nine blocks

This study was part of the Malaria Elimination Demonstration Project, which was being carried out in all 1233 villages, 297 sub-centres, and nine blocks viz*.* Niwas, Bijadandi, Narayanganj, Ghughari, Mandla, Mawai, Nainpur, Bicchiya, and Mohgaon of the Mandla district of Madhya Pradesh state in Central India (Fig. 1). The district sprawls over an area of 5800 Km^2^ with a population of 1.15 million residents and a population density of 182 people per square kilometre. Rural population constitutes around 87% of the total inhabitants with 58% classified as Scheduled Tribes (ST) and another 4.6% as Scheduled Castes (SC) [17].

The project had a staff of qualified and trained 235 Village Malaria Workers (VMWs) working pan-district. Each VMW had the responsibility of door-to-door active surveillance in 6–8 villages. Each household was visited within 7–14 days depending upon the size and population of the working area. These VMWs were supervised by 25 Malaria Field Coordinators (MFCs), who were posted at the cluster level [18]. Each MFC supervised his/her VMW using an Advance Tour Plan that was reshuffled every week. During this visit, the monitoring tool was administered by the MFC.

### Study tool

The monitoring tool (Additional file 1) was developed based on the findings from spot-inspections by district staff, qualitative feedback of the community and field staff, and feedback during monthly reviews of the malaria field coordinators (MFCs) at the district HQs. Since the tool was to be used everyday by all the MFCs, efforts were made to make it robust, easy, quick, and user-friendly. The design captured a majority of responses as categorical data viz*.* Yes/No (nominal), scoring out of five (ordinal), and numerical. The tool was divided into three sections: (1) General tool questions to be assessed by the supervisor by observing the VMW (Qs 1 to 17); (2) Interview of randomly selected fever cases tested/treated by the VMW during his/her last visit in the village (Qs 18 to 22); (3) Facts and figures (Qs 23 to 30). The last section was designed only as a refresher on facts and figures of a particular village for the respective VMW, therefore, sections one and two have been considered for data analysis for this study. The tool was pilot-tested in three blocks—Mandla, Mawai, and Niwas; which successfully captured the diverse demographics and malaria endemicity of the district. Following the pilot; the feedback was discussed, the tool was revised, re-evaluated by the experts, and rolled out pan-district.

Since this study was nested in the operational plan of MEDP. The Standard Operating Procedure (SOP) for the management of field operations was applied to all the study participants. The monitoring visits included reporting any SOP violation, including improper dress code, unapproved absence from the field, and false data reporting.

### Study duration, data collection, and analysis

The monitoring tool was administered by each of the 25 Malaria Field Coordinators for their respective VMWs on each day of field supervision. Random, unannounced, spot-inspections of the monitoring visits of the MFCs were done by MEDP district staff and Government district-staff to ensure data quality and accuracy. The MEDP district staff consisted of the Programme Officer, District Officer, and IEC/BCC Officer. The Government staff mostly consisted of the District Malaria Officer (DMO) and the District Vector Borne Disease Control Consultant (DVBDC). The tool was used as part of the regular field operations from November 2018 to February 2021. Every single tool captured data from one to three Village Malaria Workers. For analysis, all the entries were considered.

To study the effect of systematic monitoring using this tool, the timeline from November 2018 to February 2021 was divided into seven quarters (Table 1). The data was entered using CS-PRO and exported in IBM-SPSS26.0 statistical software package (IBM Corp., Armonk, NY, USA).

**Table 1:** Descriptive summary of study period divided into seven quarters with the total number of Village Malaria Workers (VMWs), number of supervisory visits, rate of visits per month, approximate population covered by the VMWs, total RDTs used, total number of positive cases, and number of new VMWs introduced every quarter

Quarter	Number of total VMWs	Number of supervisory visits	Number of VMWs monitored during supervisory visits	Number of supervisory visits per VMW per month	Number of VMWs monitored per visit	Population covered (approx.)	Number of RDTs used	Number of positive cases	Number of new VMWs
First (1 November 2018 to 28 February 2019)	212	980	1768	1.2	1.8	11,55,024	28,004	20	12
Second (1 March 2019 to 30 June 2019)	229	1848	3189	2.0	1.7	11,55,024	30,933	33	16
Third (1 July 2019 to 31 October 2019)	228	1682	2941	1.8	1.7	11,45,152	71,874	58	21
Forth (1 November 2019 to 29 February 2020)	221	1934	3378	2.2	1.7	11,10,600	37,109	44	12
Fifth (1 March 2020 to 30 June 2020)	212	1497	2754	1.8	1.8	11,35,280	23,698	31	13
Sixth (1 July 2020 to 31 October 2020)	150	629	1105	1.0	1.8	5,42,960	15,618	36	0
Seventh (1 November 2020 to 28 February 2021)	67	404	730	1.5	1.8	2,22,120	6243	4	0
Total		8974	15,865	1.8	**1.8**	6,466,160	213,479	226	74

Descriptive statistics were performed for different monitoring indicators in the tool. The variables dependent on the performance of field staff were isolated and scored against each of the nine blocks of Mandla district. These variables consisted of 11 questions with 10 questions with a maximum score of one and the last question with a maximum score of five, leading the total maximum monitoring score to be 15 (Table 2). These scores were then totaled to obtain performance scores of different blocks. These monitoring scores were further compared using ANOVA with the educational status and baseline monitoring scores, post-six months monitoring scores, post-12 months monitoring scores, post-18 months monitoring scores, and post-24 months monitoring scores. The post-training assessment scores consisted of general knowledge related to malaria (KAP—8 questions), diagnosis and treatment (DXRX—8 questions), and vector control (PVC—4 questions) adding up to a maximum scoreof 20. These scores were taken from a companion paper published by the same project [19].

**Table 2:** List of eleven questions considered for calculation of the monitoring scores along with maximum score against the possible responses. Maximum score achievable was 15

Question number (as per the monitoring checklist—Additional file 1)	Question	Response	Score	Maximum score
Q1	Was s/he found as per micro tour plan assigned	Yes	1	1
		No	0	
Q2	Does s/he know the procedure of RDT conduction and interpretation	Yes	1	1
		No	0	
Q3	Does s/he know to administer anti-malarial doses	Yes	1	1
		No	0	
Q5	Does s/he know how to read and use the expiry date on logistics?	Yes	1	1
		No	0	
Q8	Does the VMW have adequate stock of commodities and drugs	Yes	1	1
		No	0	
Q10	Are RDT kits and logistics being stored as per guidelines laid down by FDEC India?	Yes	1	1
		No	0	
Q13	Is VMW involved in source reduction for larval control or minor engineering	Yes	1	1
		No	0	
Q14	Is VMW actively involved with village health and sanitation committee	Yes	1	1
		No	0	
Q15	Is VMW engaging regularly with ASHA, ANM, panchayat leaders and local leaders?	Yes	1	1
		No	0	
Q16	Was the VMW following the dress code?			5
	a) ID card	Yes	1	
		No	0	
	b) Formal clothing	Yes	1	
		No	0	
	c) Blue apron	Yes	1	
		No	0	
	d) Bag	Yes	1	
		No	0	
	e) Groomed	Yes	1	
		No	0	
Q17	Check VMW’s bag, is s/he carrying any non-project supplies? Exempt water bottle and lunch box	Yes	0	1
		No	1	
	Maximum monitoring score			15

## Results

The VMWs were monitored using the monitoring tool with a total of 8,978 visits consisting of 15,886 observations between November 2018 to February 2021 (Table 1). During the visits, each MFC supervised at least one VMW, followed by two VMWs by 76% MFCs, and very few could supervise three VMWs in a single visit (1.4%). On average, each VMW was supervised 1.8 times in a month by his/her respective MFC.

The critical monitoring indicators: (1) adherence to the Advance Tour Plan; (2) knowledge of Rapid Diagnostic Test (RDT) procedure and interpretation; (3) knowledge of anti-malarial drug regimens; (4) submission of daily work-done reports; (5) proper handling of anti-malaria drugs including supply chain achieved an average of more than 99% scores through all the seven quarters, respectively. Dress code adherence of VMWs was scored an average of 4.3/5 by the supervisors throughout the study period and only 3.9% VMWs were found carrying non-project supplies in their bag packs during field visits. These defaulters decreased to zero percent in the last two-quarters of the study period (Table 3).

**Table 3:** Mean monitoring scores of different blocks of Mandla district

Indicators/blocks	Mean monitoring scores									
	Bichhiya	Bijadandi	Ghughri	Mandla	Mawai	Mohgaon	Nainpur	Naray anganj	Niwas	Total
1. Adherence to Advance Tour Plans (ATPs)	0.99	1.00	0.98	0.99	1.00	0.98	1.00	0.99	0.99	0.99
2. Knowledge of RDT conduction and interpretation	1.00	1.00	0.99	1.00	1.00	0.99	1.00	0.99	0.99	1.00
3. Knowledge of anti-malarial dosages	0.99	1.00	0.98	0.98	0.99	0.99	1.00	0.98	0.99	0.99
4. Daily reporting	1.00	1.00	1.00	1.00	1.00	1.00	1.00	0.99	0.99	1.00
5. Proper handling of drugs and stocks	1.00	1.00	0.99	1.00	1.00	1.00	1.00	1.00	0.99	1.00
6. Maintenance of adequate stock	0.95	0.66	0.99	0.98	0.90	1.00	0.97	0.98	0.96	0.93
7. Proper storage of commodities	0.99	0.98	1.00	0.99	0.98	1.00	0.99	0.99	0.97	0.99
8. Involvement in source reduction	0.81	0.49	0.93	0.94	0.52	0.82	0.41	0.61	0.84	0.71
9. Involvement with Village Health and Sanitation Committee	0.05	0.20	0.77	0.19	0.27	0.23	0.20	0.23	0.05	0.23
10. Inter-sectorial coordination with local ASHAs	0.93	0.82	0.97	0.97	0.96	0.94	0.96	0.92	0.93	0.94
11. Rating of field staff by the beneficiary	3.74	3.96	3.90	3.04	3.75	3.97	3.94	3.73	3.34	3.68
Total Monitoring Score (out of 15)	12.46	12.11	13.49	12.07	12.36	12.92	12.47	12.42	12.04	12.45

(Indicators from one to ten had a maximum score of one and the eleventh indicator had a maximum score of five. Total monitoring scores have been calculated from a maximum score of 15)

The MEDP staff visited the Bicchiya, Mawai, and Mandla blocks the highest number of times as compared to any other blocks. The monitoring visits were highest in the first quarter (1 November 2018 to 28 February 2019) by the Government officials (2%) and MEDP district staff (12.3%) showing statistically significant difference as compared to other quarters. However, the supervisory visits by the MFCs were consistent throughout the study period with no statistically significant difference between the quarters. The inadequate stock of anti-malarial and diagnostics was noted for an average of 6.7% by VMWs throughout the study period with only 2.2% VMWs during the high transmission season.The proper storage and handling of diagnostics, medicines, and supplies by the VMWs increased from 93.3% in the first quarter to 98.5% in the second quarter and stabilized at 99.7% for the remaining quarters.

Involvement of the VMWs in source reduction of mosquito larvae through minor engineering was found to be highest during transmission seasons in 2019 and 2020 (July to October) at 84.4% and 94%, respectively. In the remaining months, the involvement averaged at only 67%. The highest involvement was seen in the urban areas of Mandla (93.9%) and Ghughari blocks (92.6%) with the least in rural areas of Nainpur (41%) and Mawai (52.2%) blocks. At 20.3%, low involvement of VMWs was observed with the Village Health and Sanitation Committee. A good level of collaboration (94%) by engaging with the local Accredited Social Health Activists (ASHAs) was seen throughout the study period.

The average time taken by the VMWs after interpretation of RDT results and the start of treatment was 8 min with the highest in Mandla block (13 min) and lowest in Niwas and Bicchiya blocks (5 min). Statistically, a significant difference was noted in progressively increasing diagnosis to treatment time over the study period and scoring of VMW’s behavior by the patient.

The mean monitoring scores of different blocks showed the highest score by Ghughari block (12.93) followed closely by Mohgaon (12.88) and Nainpur (12.47). The lowest scoring block was Niwas (12.01), however, no statistically significant difference was observed between these scores. The comparison of the scores of VMWs with their post-training scores and educational status (Table 4) and the duration of monitoring against their scores did not yield any statistically significant difference (Table 5).

**Table 4:** Association of educational level of the Village Malaria Workers with their baseline monitoring scores (maximum score = 15), and post-training scores (maximum score = 20) at post-six months monitoring scores, post-12 months monitoring scores, post-18 months monitoring scores, and post-24 months monitoring scores

Education level	Training scores	Baseline monitoring scores	Post-six months monitoring scores	Post-12 months monitoring scores	Post-18 months monitoring scores	Post-24 months monitoring scores
12th						
Mean	16.9	12.0	12.8	12.4	11.9	11.8
Median	17.0	12.0	13.0	13.0	12.0	12.0
Std. Deviation	2.4	1.5	1.4	1.5	1.8	1.7
N	76	76	57	56	50	6
Graduation						
Mean	17.7	11.6	12.5	12.4	12.0	13.0
Median	18.0	12.0	13.0	13.0	12.0	13.0
Std. Deviation	1.8	2.0	1.3	1.4	1.7	1.1
N	163	163	130	113	99	20
Post-graduation						
Mean	17.3	11.2	12.4	12.3	11.4	13.0
Median	17.0	12.0	13.0	13.0	12.0	13.0
Std. Deviation	1.7	1.9	1.3	1.3	1.7	0.7
N	48	48	37	29	25	5
Total						
Mean	17.4	11.6	12.6	12.4	11.9	12.7
Median	18.0	12.0	13.0	13.0	12.0	13.0
Std. Deviation	2.0	1.9	1.3	1.4	1.7	1.2
N	287	287	224	198	174	31
ANOVA (F)	4.0	2.7	1.0	0.0	1.3	2.3

*p < 0.05

**Table 5:** A comparison of Village Malaria Workers’ baseline monitoring scores (Maximum score = 15) and post-training scores (maximum score = 20) at post-six months monitoring scores, post-12 months monitoring scores, post-18 months monitoring scores, and post-24 months monitoring scores by training their scores (grouped in three categories viz. less than or equal to 16, between 17 to 18, and between 19 to 20)

Training score groups	Training scores	Baseline monitoring scores	Post-six months monitoring scores	Post-12 months monitoring scores	Post-18 months monitoring scores	Post-24 months monitoring scores
≤16						
Mean	14.8	11.7	12.5	12.4	11.7	12.7
Median	15.5	12.0	13.0	13.0	12.0	13.0
Std. Deviation	1.5	2.0	1.4	1.2	1.7	1.6
N	82	82	70	61	53	11
17–18						
Mean	17.6	11.7	12.7	12.3	11.9	12.6
Median	18.0	12.0	13.0	13.0	12.0	13.0
Std. Deviation	0.5	1.9	1.3	1.5	1.7	1.1
N	109	109	90	90	75	13
19–20						
Mean	19.4	11.5	12.5	12.6	12.1	13.0
Median	19.0	12.0	13.0	13.0	12.5	13.0
Std. Deviation	0.5	1.7	1.3	1.3	1.9	0.6
N	96	96	64	47	46	7
Total						
Mean	17.4	11.6	12.6	12.4	11.9	12.7
Median	18.0	12.0	13.0	13.0	12.0	13.0
Std. Deviation	2.0	1.9	1.3	1.4	1.7	1.2
N	287	287	224	198	174	31
ANOVA (F)	592.5*	0.5	0.7	0.4	0.8	0.2

*p < 0.05

## Discussion

The study design [18] and companion studies of the Malaria Elimination Demonstration Project focusing on malaria epidemiology [20], vector control [21], entomology [22], and capacity building [19] have emphasized the importance of robust monitoring and supervision to achieve malaria elimination goals. This study investigated the use of a structured monitoring tool for field staff performing active surveillance and case management, vector control, IEC and BCC, and capacity-building activities.

Since the start of its field operations in September 2017, the project followed an open-ended qualitative monitoring mechanism for better understanding the capacity of the field staff of the project. These learnings were incorporated in the development of the monitoring tool. A similar strategy was followed while developing the IEC and BCC strategy of the project [15, 18]. The systematic process of developing a structured tool to gain high-fidelity work-products has been recommended by Tolley [23], Baranowski [24], and Resnicow [25]. Monitoring tools have been recommended by the WHO [2] and the National Vector Borne Disease Control Programme for the implementation of malaria elimination programmes [26]. This study has reinforced the value of the monitoring tool developed based on the results of the pilot study.

As per the operational plan of MEDP, each MFC was supposed to do in-person monitoring of each of his/her VMW areas at least 2 times a month. The total number of monitoring visits reduced in the last two quarters as MEDP initiated the phased closure of its field operations from June 2020 to March 2021. Efforts were made to maintain a uniform surveillance strategy for the entire district and not to be affected by the status of malaria-endemicity of any particular block. However, different blocks posed different characteristics viz*.* Mawai, Bicchiya, and Ghughari were most malaria-affected; Mandla and Nainpur were pre-dominantly urban; Narayanganj and Mohgaon had a maximum number of hard-to-reach areas, and Niwas and Bijadandi were the farthest from the district HQs [17]. Relatively higher monitoring in Bicchiya and Mawai was because of higher malaria endemicity in these blocks. High malaria endemicity led to frequent patient follow-up visits by the MFCs in addition to the regular monitoring visits. Mandla block is the district HQs and the increased visits by the MFCs were made to reinforce the inter-personal communication to gain maximum compliance from key-opinion leaders of the community. The Mohgaon, Narayanganj, and Niwas blocks had the least monitoring visits due to hard-to-reach areas and distance from the district HQs, which also led to frequent vacant VMW positions.

High scores in adherence to advance tour plans, knowledge of RDT procedures and interpretation, knowledge of anti-malarial drug regiments, submission of daily work-done reports, proper handling of anti-malarial drugs were observed since the beginning of the study. This was because of MEDP’s strong focus on these indicators since the beginning of the field operations [15, 18]. Additionally, the mobile application surveillance tool (SOCH) was introduced in July 2018, which further improved the overall accountability, reporting, and supply chain management systems [27].

In this study, the adequate stock was available in 93.3% of VMW areas during the entire year and 97.8% in the high-transmission season. It was observed that despite using the Solutions for Community Healthworkers (SOCH) mobile application [27], few situations of medicines and diagnostics stock-outs arose due to interruption in raising of stock requests by the VMWs. These interruptions included human error, mobile network, and hardware issues. Similar situations with varying magnitudes were also observed in an ASHA needs-assessment study done in the study area [28]. Another study carried out in Eastern Myanmar reported that 14% of the malaria posts had inadequate stocks [29].

As part of MEDP activities, the collaboration with the local ASHA workers was emphasized. This collaboration included real-time sharing of information and training of the ASHAs based on the findings of the needs assessment study conducted by the project [28]. Involvement of the VMWs in source reduction using minor engineering peaked during transmission seasons. The minor engineering usually involved draining stagnant water from discarded tires, stray containers, puddles, water coolers, and covering of open water storage tanks. The incidence of stagnant water collides with the advent of rains during the transmission season, hence, a similar pattern was captured during the monitoring visits. This issue has been documented by companion studies of MEDP [17, 19, 30] and others in the same district [31].

Most minor engineering activities were done in the urban areas of Mandla and Ghughari, which may be attributed to better socio-economic characteristics, including better awareness and more household commodities such as water coolers or flower pots [17]. A similar reason of higher education and awareness of the residents may be the reason for the increased gap between RDT interpretation and the start of treatment by the VMWs in Mandla block, which is predominantly urban. It is common in such places of higher education for patients to seek second opinion and asking more questions as compared to other blocks before initiating the treatment. Similar findings were reported by the urban vs. rural General Practioners of Germany [32].

No significant difference was noted between monitoring scores of different blocks, educational status of VMWs, training scores,and monitoring scores. It was interesting to note that there was no effect of educational status (12th vs. graduation vs. post-graduation) on the monitoring scores. This observation from MEDP suggests that the success of malaria elimination field-work would depend on properly trained field staff and not staff that holds higher educational degrees. Similar observation was made by Abhay Bang et al., working in another tribal district of Gadchiroli on reduction of pneumonia and total childhood mortality [33].

The lack of progressive improvement in the monitoring scores over 24 months indicated a need for continuous and innovative training and challenges that will help the field staff in improving their professional capabilities. In the training assessment study of MEDP, it was observed that after continuous monitoring and training of the field staff, the post-assessment scores leveled at par with the maximum scores [19]. A limitation of the study was that the exhaustive monitoring tool used in this study proved challenging to be used on a daily basis. The authors recommend that monitoring checklists should be lean to optimize efficiency and reduce fatigue in the field staff.

## Conclusion

This study demonstrated the use of a robust monitoring tool to assess the performance and activities of the field staff working at the ground level. MEDP achieved a reduction in indigenous malaria cases by 91%, which was in-part attributed due to robust monitoring and training of the field staff. The study has also highlighted the need for continuous training of field staff for improving the performance of the field staff. Close monitoring of the logistics and prevention of stock-outs enabled the field staff to deliver their duties more efficiently. These observations suggest that similar tools should be adapted in national or sub-national malaria elimination and other public health programmes to achieve timely, reliable, and replicable results.

## Supplementary Information

**Additional file 1:** Checklist for monitoring and evaluation.

## Data Availability

We have reported all the findings in this manuscript. The hardcopy data is stored at MEDP Office in Jabalpur, Madhya Pradesh, and Indian Council of Medical Research-National Institute of Research in Tribal Health (ICMR-NIRTH), Jabalpur, Madhya Pradesh. Softcopy data is available on the project server of MEDP hosted by Microsoft Azure. If anyone wants to review or use the data, they should contact: Dr. Altaf A. Lal. Project Director—Malaria Elimination Demonstration Project, Mandla. Foundation for Disease Elimination and Control of India, Mumbai, India 482,003. E mail: altaf.lal@sunpharma.com, altaf.lal@gmail.com.

## Abbreviations

ALMA: Africa Leaders Malaria Alliance
ANOVA: Analysis of Variance
API: Annual Parasitic Incidence
ASHA: Accredited Social Health Activist
ATP: Advance Tour Plan
CS PRO: Census and Survey Processing System
GoMP: Government of Madhya Pradesh
HQs: Headquarters
IBM: International Business Machines
ICMR: Indian Council of Medical Research
MEDP: Malaria Elimination Demonstration Project
MFC: Malaria Field Coordinator
NIRTH: National Institute of Research in Tribal Health
NVBDCP: National Vector Borne Disease Control Programme
NY: New York
RDT: Rapid Diagnostic Test
SC: Scheduled castes
SOCH: Solutions for Community Health-workers
SPSS: Statistical Package for Social Sciences
ST: Scheduled tribes
VMW: Village Malaria Worker
