# Supplementary material for: Monitoring of the Village Malaria Workers to conduct activities of Malaria Elimination Demonstration Project in Mandla, Madhya Pradesh
Source: Malar J. 2022 Jan 8;21:18. doi: 10.1186/s12936-021-04040-2 (PMC8742915; doi:10.1186/s12936-021-04040-2)
Supplement: Supplementary file 1 — Additional file 1: Checklist for monitoring and evaluation. [file 12936_2021_4040_MOESM1_ESM.pdf]

Form 3: Checklist for Monitoring and Evaluation

Foundation for Disease Elimination and Control of India (FDEC-I)
Mandla- Malaria Elimination Demonstration Project, (M-MEDP)
Madhya Pradesh
Malaria Field Coordinator (MFC) checklist for Village Malaria Worker (VMW)

Date: DD/MM/YY Block .... Cluster.. Name of MFC ....

	Name of employee	Employee ID	Name of visited village with code
VMW1			
VMW 2			
VMW 3			

	Checklist points	VMW 1	VMW 2	VMW 3
1	Was s/he found as per micro tour plan assigned	(YES/NO)	(YES/NO)	(YES/NO)
2	Does s/he know the procedure of RDT conduction & interpretation	(YES/NO)	(YES/NO)	(YES/NO)
3	Does s/he know to administer anti-malarial doses (ask few random question)	(YES/NO)	(YES/NO)	(YES/NO)
4	When did VMW submit the DAR in mobile app (last working day report) mention date	(YES/NO)	(YES/NO)	(YES/NO)
5	Did he know how to read and use the expiry date on logistics?	(YES/NO)	(YES/NO)	(YES/NO)
6	Write the code number of last blood sample collected on filter paper and glass slide	(NUMBER)	(NUMBER)	(NUMBER)
7	No. of visits made last week/ last month to supervise the work of VMW by the following:	-	-	-
	• GoMP (mention name and designation of official here: ______________________)	(NUMBER)	(NUMBER)	(NUMBER)
	• FDEC India (mention name and designation of official here: ______________________)	(NUMBER)	(NUMBER)	(NUMBER)
	• MFC (mention name here: ______________________)	(NUMBER)	(NUMBER)	(NUMBER)
8	Does the VMW have adequate stock of commodities & drugs (RDT, ACT, CQ,PQ, Filter paper etc.)	(YES/NO)	(YES/NO)	(YES/NO)
9	Are there any drugs at risk of expiry with in next 3 months (physically verify). If yes, mention here: ______________________	(If yes, mention/NO)	(If yes, mention/NO)	(If yes, mention/NO)
10	Are RDT kits and logisticsbeing stored as per guidelines laid down by FDEC India?	(YES/NO)	(YES/NO)	(YES/NO)
11	No. of patient refer to health facility by VMW in last week/month for non-malarial patient	(YES/NO)	(YES/NO)	(YES/NO)
12	No. of patient refer to health facility by VMW in last week/month for malaria positive patient	(YES/NO)	(YES/NO)	(YES/NO)
13	Is VMW involved in source reduction for larval control or minor engineering	(YES/NO)	(YES/NO)	(YES/NO)
14	Is VMW actively involved with village health and sanitation committee	(YES/NO)	(YES/NO)	(YES/NO)
15	Is VMW engaging regularly with ASHA, ANM, Panchayat and local leaders? Cross check with partners.	(YES/NO)	(YES/NO)	(YES/NO)
16	Was the VMW following dress code (ID card, formals, blue apron, bag, groomed) – Give 1 mark for each	/5	/5	/5
17	Check VMW's bag – Is he carrying any non-project supplies? (Exempt – Lunch box, water bottle etc.) If yes, mention here:	(YES/NO)	(YES/NO)	(YES/NO)
	**Interview of randomly selected fever cases tested/treated by VMW in the last visit at this village. Mention date of last visit : DD/MM/YY**			
18	Did VMW test the patient by RDT	(YES/NO)	(YES/NO)	(YES/NO)
19	What was time difference between test done and treatment start (mention in minutes /hours)	(NUMBER)	(NUMBER)	(NUMBER)
20	Was any drug given to the malaria negative patient? If yes, which and how much? ______________	(YES/NO)	(YES/NO)	(YES/NO)
21	Was money charged for the test/treatment	(YES/NO)	(YES/NO)	(YES/NO)
22	How would you rate the VMWon his behavior: (1 being the worst and 5 being excellent)(Was he soft spoken? Did he take your consent before testing?)	/5	/5	/5
	**Fact and figures**			
23	Total number of fever cases tracked by VMW in the last 4 weeks/one month	(NUMBER)	(NUMBER)	(NUMBER)
24	No. of malaria positive cases (PF+PV+MIX=Total cases) during this week, month and year	Wk/Mo/Yr	Wk/Mo/Yr	Wk/Mo/Yr
25	No. of cases undergoing / completed radical treatment during this week, month and year	Wk/Mo/Yr	Wk/Mo/Yr	Wk/Mo/Yr
26	No. of live birth/ death in village during this week, month and year	Wk/Mo/Yr	Wk/Mo/Yr	Wk/Mo/Yr
27	No. of call attended from fever patient in the past week	(NUMBER)	(NUMBER)	(NUMBER)
28	No. of household survey conducted (Count households)	(NUMBER)	(NUMBER)	(NUMBER)
29	No. of LLIN verified during his/her visit during this week, month and year	Wk/Mo/Yr	Wk/Mo/Yr	Wk/Mo/Yr
30	No. of timesparticipated in IEC/ BCC campthis week, month and year (School + Haat bazaar)	Wk/Mo/Yr	Wk/Mo/Yr	Wk/Mo/Yr

Additional observations: ....

....

Name and Signature of MFC
